# Clinical utility of diaphragmatic ultrasound for mechanical ventilator liberation in adults: a systematic review and meta-analysis

**DOI:** 10.1186/s40560-025-00811-0

**Published:** 2025-07-24

**Authors:** Naonori Tashiro, Hiroki Nishiwaki, Takashi Ikeda, William M. M. Levack, Hisashi Noma, Noyuri Yamaji, Erika Ota, Takeshi Hasegawa

**Affiliations:** 1 Division of Cardiopulmonary Rehabilitation Science, Department of Rehabilitation, Showa Medical University Graduate School of Health Sciences, 1865 Tokaichibacho, Midori-ku, Yokohama-shi, Kanagawa 226-8555 Japan; 2 Institute of Clinical Epidemiology (iCE), Showa Medical University, 1-5-8 Hatanodai, Shinagawa-Ku, Tokyo 142-8555 Japan; 3 Internal Medicine (Nephrology), Internal Medicine Center, Showa Medical University Fujigaoka Hospital, 1-30 Fujigaoka, Aoba-ku, Yokohama-shi, Kanagawa 227-8501 Japan; 4 Rehabilitation Center, Showa Medical University Fujigaoka Rehabilitation Hospital, 2-1-1 Fujigaoka, Aoba-ku, Yokohama-shi, Kanagawa 227-8518 Japan; 5 Division of Clinical Nutrition, Department of Medical Basics, Specialty and Education, Showa Medical University Graduate School of Health Sciences, 1865 Tokaichibacho, Midori-ku, Yokohama-shi, Kanagawa 226-8555 Japan; 6 Department of Medicine, University of Otago, Wellington, Mein St, Newtown, PO Box 7343, Wellington, 6242 Aotearoa New Zealand; 7 Department of Data Science, The Institute of Statistical Mathematics, 10-3 Midoricho, Tachikawa-shi, Tokyo 190-8562 Japan; 8 Global Health Nursing, St. Luke’s International University Graduate School of Nursing, 10-1 Akashicho, Chuo-ku, Tokyo 104-0044 Japan; 9 Department of Hygiene, Public Health, and Preventive Medicine, Showa Medical University Graduate School of Medicine, 1-5-8 Hatanodai, Shinagawa-Ku, Tokyo 142-8555 Japan; 10 Department of Nephrology, Showa Medical University Graduate School of Medicine, 1-5-8 Hatanodai, Shinagawa-Ku, Tokyo 142-8555 Japan; 11 Showa Medical University Research Administration Center, Showa University, 1-5-8 Hatanodai, Shinagawa-Ku, Tokyo 142-8555 Japan; 12 Center for Innovative Research for Communities and Clinical Excellence, Fukushima Medical University, 1 Hikarigaoka, Fukushima City, Fukushima 960-1295 Japan; 13 Department of Healthcare Epidemiology, School of Public Health in the Graduate School of Medicine, Kyoto University, Yoshida Konoe-cho, Sakyo-ku, Kyoto, 606-8501 Japan

**Keywords:** Ventilator liberation, Ultrasound, Diaphragmatic dysfunction, Mechanical ventilation, Critical care

## Abstract

**Background:**

Prolonged mechanical ventilation is associated with an increased incidence of complications and higher mortality rates. Therefore, it is crucial to wean patients from mechanical ventilation as soon as possible. Recently, diaphragmatic ultrasound has been used in this decision-making process. This systematic review evaluated the effectiveness of diaphragmatic ultrasound to improve ventilator liberation outcomes.

**Methods:**

We searched three databases – MEDLINE, Embase, and the Cochrane Central Register of Controlled Trials. We included randomized control trials that compared the use of diaphragmatic ultrasound to standard care in adult patients on mechanical ventilation via tracheal intubation. We assessed risk of bias for included trials with the Cochrane Risk of Bias Tool and certainty of evidence using the Grading of Recommendations, Assessment, Development, and Evaluation tool. For dichotomous outcomes, we reported risk ratios (RRs) with 95% confidence intervals (CIs). For continuous outcomes, we reported mean differences (MD) with 95% CIs if all retrieved records provide data on the same scale. The primary outcome was incidence of reintubation within 48 h of extubation and the secondary outcomes included duration of mechanical ventilation, incidence of reintubation rate after 48 h, ICU length of stay, and adverse events.

**Results:**

We found five relevant randomized controlled trials involving a total of 508 participants on mechanical ventilation in ICU following respiratory failure or surgery. Three studies (268 participants) provided data on the incidence of reintubation within 48 h of extubation. Using diaphragmatic ultrasound to guide extubation decisions led to a significant reduction in the risk of reintubation within 48 h (RR 0.62, 95% CI 0.41 to 0.95, low certainty of evidence). No significant differences were found in the duration of mechanical ventilation (MD − 1.39 h, 95% CI − 17.5 to 14.71 h, three studies, 268 participants, very low certainty of evidence) or reintubation after 48 h (RR 0.38, 95% CI 0.11–1.29, two studies, 240 participants, moderate certainty of evidence). However, ICU length of stay was significantly reduced in the diaphragmatic ultrasound group (MD − 1.0 days, 95% CI − 1.74 to − 0.26 days, one study, 130 participants, low certainty of evidence).

**Conclusion:**

Using diaphragmatic ultrasound in addition to standard clinical criteria to guide decisions around ventilator use and liberation resulted in a reduced risk of reintubation within 48 h of extubation when compared to standard clinical criteria alone.

**Systematic review registration:**

This systematic review was registered with the Open Science Framework: https://osf.io/cn8xf.

**Supplementary Information:**

The online version contains supplementary material available at 10.1186/s40560-025-00811-0.

## Background

Diaphragmatic dysfunction has been identified in 60% to 80% of people on mechanical ventilation, most likely resulting from diaphragm atrophy, sepsis, or systemic infection [1]. Diaphragmatic dysfunction occurs twice as frequently as limb weakness among people on mechanical ventilation and has been associated with increased risk of ventilator weaning failure [2]. Prolonged weaning is of particular concern because of its association with increased mortality. Patients who fail three or more liberation attempts or who require more than seven days to wean after a first attempt have a higher risk of in-hospital death compared to people who achieve ventilation liberation on their first attempt without difficulty. One study reported an odds ratio of 4.89 (95% confidence interval (CI) 1.32–18.08) for in-hospital death associated with prolonged compared to simple weaning [3]. Based on these points, prolonged mechanical ventilation is associated with an increased incidence of complications and higher mortality rates.

More recently, evaluation of diaphragm function by ultrasound has been used to predict readiness for ventilator liberation, with diaphragm thickening fraction (DTF), diaphragmatic excursion (DE), and the diaphragmatic rapid shallow breathing index (DRSBI) being used to predict success of ventilator liberation [4–7]. Systematic reviews published to date on the use of diaphragmatic ultrasound during ventilator liberation have been based on assessment of diagnostic accuracy [8]. There have been no systematic reviews that have pooled data from randomized controlled trials (RCTs), and it is unclear whether adding diaphragmatic ultrasound to the standard criteria results in differences in subsequent outcomes. Therefore, the purpose of this systematic review of RCTs was to evaluate the effectiveness of diaphragmatic ultrasound to guide the decision-making process for ventilator withdrawal in intensive care unit (ICU) settings.

## Methods

This systematic review followed the Preferred Reporting Items Statement for Systematic Reviews and Meta-Analyses (PRISMA) [9] and its protocol was registered on the Open Science Framework Registries (OSF) website (https://osf.io/cn8xf). We published the full protocol for this review prior to beginning study selection and data extraction [10].

### Criteria for considering studies for this review

#### Types of studies

We included RCTs, both individually randomized trials and cluster randomized trials. Studies without a standard criteria comparison group were excluded. Case series, case reports, editorials, letters to editor, and conference abstracts were also excluded from this review.

#### Types of participants

We included studies with adult patients (≧18 years old) who had been intubated and were managed by mechanical ventilation in ICU.

#### Types of interventions

We included all studies that tested the use of diaphragmatic ultrasound to guide clinical decision-making regarding extubation, including studies that tested the use of diaphragmatic ultrasound to assess readiness for extubation or tolerance for a spontaneous breathing trial (SBT). We included studies using any of the following ultrasound assessment methods:

- Measurement of diaphragmatic movement, using DE,
- Assessment of the change in diaphragmatic thickness, using DTF
- Evaluation of respiratory effort during a single breath, using DRSBI.

### Types of outcome measures

#### Primary outcomes

The primary outcome was the incidence of reintubation within 48 h of extubation. Studies that had other thresholds, such as reintubation within 72 h of extubation, or did not allow for reintubation within 48 h were excluded from this analysis.

#### Secondary outcomes

Secondary outcomes included the following:

1. Total duration of mechanical ventilation
2. Reintubation after 48 h
3. Total ICU length of stay, and
4. Adverse events, specifically the frequency of infections arising from cross-contamination during ultrasound procedures

### Search methods for identification of studies

#### Electronic searches

To identify eligible trials, we searched MEDLINE, Embase, and Cochrane Central Register of Controlled Trials (CENTRAL) from inception to 1 April 2025. We limited this review to human studies published in English. We designed our search strategy based on advice from a librarian experienced in conducting systematic reviews. Search terms included the MeSH and keywords associated with respiration, ventilator liberation, diaphragm, and sonography (Additional file 1: Table S1).

### Data collection and analysis

#### Selection of studies

We imported all titles and abstracts into Endnote, a citation management software package, and removed duplicates. Two review authors (NT and TI) screened the titles and abstracts to identify potentially relevant articles that met our inclusion criteria. Final decisions regarding the possible relevance of each article were made through consensus with the two reviewers. Disagreements were resolved through discussion with a third author (TH). Two review authors (NT and TI) then screened the full-text publications of all potentially relevant articles before making a final decision on inclusion – again with involvement of a third author (TH) to resolve disagreements when needed. We summarized the search results in a PRISMA flow diagram.

#### Data extraction and management

For all included studies, we extracted data on study characteristics, study methods, participant characteristics, and relevant outcome data. In addition, for each study outcome, we extracted information on the definition, type, and rating of the outcome, as well as the amount of and reasons for missing data. We summarized study characteristics using frequencies and percentages for categorical variables and means and standard deviations or medians and interquartile ranges for continuous variables, depending on the data distribution.

#### Assessment of risk of bias in included studies

We used Version 2 of the Cochrane Risk of Bias tool for randomized trials (RoB 2) to assess risk of bias in each included study, separately for each outcome [11]. Two authors (NT and HN) independently conducted the assessments for each included study, with a third author (TH) resolving any disagreements. We evaluated risk of bias arising from the randomization process, deviations from the intended interventions, missing outcome data, measurement of the outcome, and selection of the reported result. We classified studies as low risk of bias (little to no concerns), moderate risk of bias (some concerns), or high risk of bias (serious concerns) in each domain.

#### Measures of treatment effect

We used intention‐to‐treat data to calculate treatment effects. For dichotomous outcomes, we calculated risk ratios (RRs) with 95% CIs. For continuous outcomes, we measured the mean difference (MD) with 95% CIs if all retrieved records provide data on the same scale.

#### Assessment of heterogeneity

We evaluated clinical and methodological heterogeneity of included studies (e.g., design features, population characteristics, the purpose of ultrasound, and intervention details) and summarize this information in a table of study characteristics. Forest plots were inspected to describe the direction and magnitude of effects and the degree of overlap of CIs. We also examined statistical heterogeneity using the estimated Cochrane Chi-square test, Tau^2^, and I^2^ statistic.

#### Statistical analysis

We performed data synthesis analysis using the DerSimonian–Laird type random-effects model [12]. We used Review Manager (RevMan) software, version 5.4 (Cochrane Collaboration) for data analysis.

#### Assessment of certainty of evidence: GRADE approach

We used the Grading of Recommendations, Assessment, Development, and Evaluation (GRADE) tool to assess the overall strength of the evidence and rated the quality of evidence for each finding based on evaluation of risk of bias, indirectness, inconsistency, imprecision, and other considerations [13]. Two investigators (NT and HN) independently rated each finding against the GRADE criteria and scored overall certainty of evidence for each finding as being high, moderate, low, or very low. A third and fourth author (NY and TH) resolved discrepancies between these two reviewers when needed. We used GRADEpro GDT to produce a Summary of Findings table highlighting the main findings and the certainty of evidence in these results.

## Results

### Search results

**Fig. 1:**
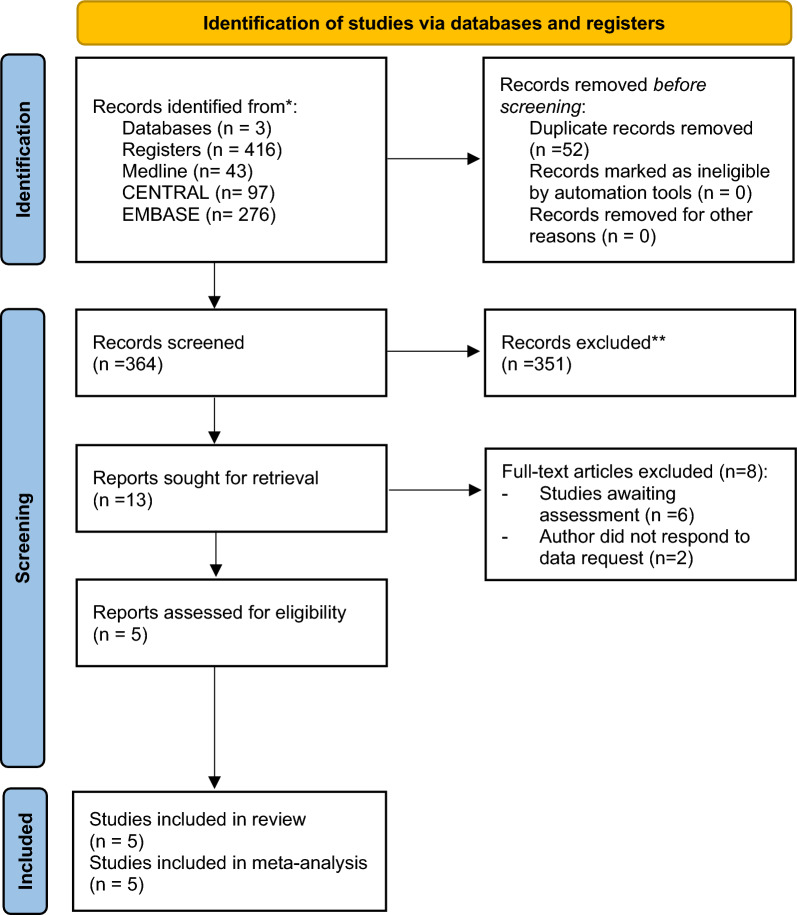
Flow diagram of the study selection

We identified 364 studies from the electronic database after eliminating duplicates. We excluded 351 of these studies based on screening of titles and abstracts, leaving thirteen studies for full-text screening. Of these thirteen studies, we excluded six studies as awaiting assessment because a full report with study results had not yet been published [14–19]. We attempted to contact the corresponding authors of two studies to obtain additional information, but no response was received [20, 21]. We therefore included five RCTs in this review [22–26] (Fig. 1).

### Study characteristics

A total of 508 patients were analyzed from five RCTs reported by Allam [22] in 2023, Toledo et al. [23] in 2023, McCool et al. [24] in 2020, Alansary et al. [25] in 2020, and Mowafy et al. [26] in 2019 (Table 1). Three of these RCTs (268 participants total, 135 in the diaphragmatic ultrasound groups, 133 in the standard criteria groups) provided data on the primary outcome (reintubation within 48 h of extubation). The study by Allam 2023 provided data on reintubation within 72 h of extubation and at study end, but not within 48 h of extubation, so was excluded from the analysis of the primary outcome. The study by Toledo 2023 did not provide data on the primary outcome because there were no patients in that study requiring reintubation within 48 h of extubation. Only one trial employed a multi-center design (McCool et al.), which incorporated data from three different hospitals within the same university. No studies reported on the frequency of infections arising from cross-contamination during ultrasound procedures.

**Table 1:**
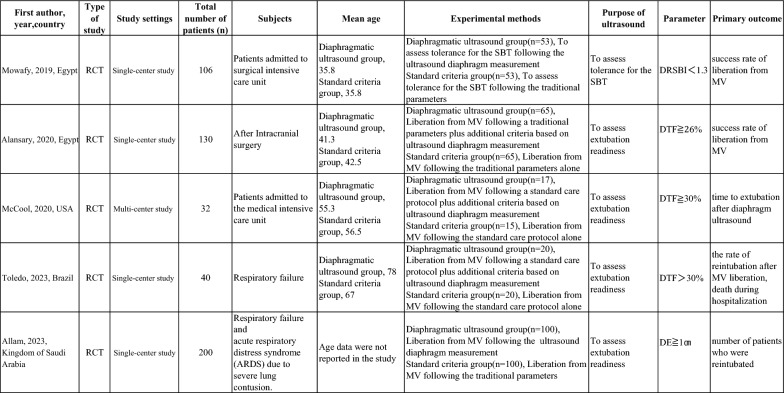
Description of all included studies

*RCT* randomized control trial, *MV* mechanical ventilation, *SBT* spontaneous breathing trial, *DTF* diaphragm thickness fraction, *DRSBI* diaphragmatic rapid shallow breathing index, *DE* diaphragmatic excursion

The included studies differed in terms of how diaphragmatic ultrasound was used to inform clinical decisions. In the study by Mowafy et al., ultrasonography was performed to assess tolerance during an SBT and was not directly used to guide extubation decisions. In contrast, in the studies by Toledo et al., McCool et al., Alansary et al., and Allam, diaphragmatic ultrasound was used to determine extubation readiness – that is, to assist in the decision-making process for liberation from mechanical ventilation (Table 1).

### Risk of bias assessment

**Fig. 2:**
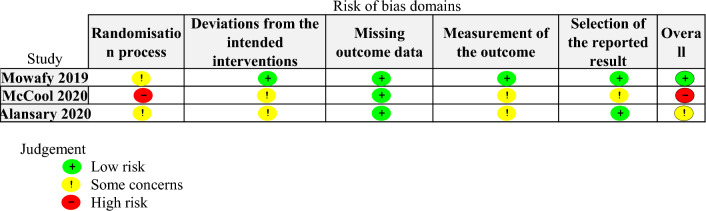
Summary of risk of bias for reintubation within 48 h

Figure 2 summarizes the risk of bias for the primary outcome in the included studies. One RCT was rated as high risk of bias due to problems with the randomization process [24]. In this study, it was unclear whether concealment of group allocation was maintained until participants were enrolled and assigned to the experiment or comparison groups [24]. The other two studies were at moderate risk of bias [25] and low risk of bias [26]. We conducted a risk of bias assessment by outcome, but the results were the same for all outcomes (Additional file 2: Figure S1).

### Primary outcome

**Fig. 3:**
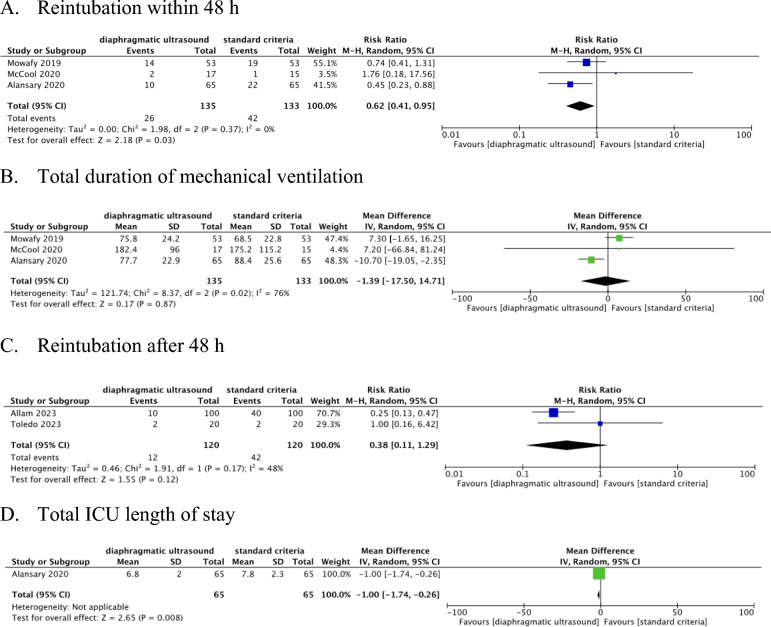
Forest plot of the diaphragmatic ultrasound and standard criteria comparison. Reintubation within 48 h (**A**), Total duration of mechanical ventilation (**B**), Reintubation after 48 h (**C**), Total ICU length of stay (**D**). No studies evaluated the effect of adverse event. *CI* confidence interval, *df* degrees of freedom, *M-H* Mantel–Haenszel test, *IV* inverse variance, *RR* risk ratio

Data on reintubation within 48 h of extubation were available from three studies [24–26]. Among the 268 patients included in these studies, 19.3% (26 out of 135 patients) in the diaphragmatic ultrasound group and 31.6% (42 out of 133 patients) in the standard criteria group were reintubated within 48 h of extubation (Fig. 3). The relative risk (RR) was 0.62 (95% CI 0.41 to 0.95) in favor of using diaphragmatic ultrasound to guide extubation decisions. No statistical heterogeneity was observed for the primary outcome (*I*^2^ = 0%, *χ*^2^ = 1.98, *P* = 0.37). The GRADE assessment (Table 2) indicated that our certainty in this finding was low. We downgraded this finding by two levels because of a) indirectness – as one study, Mowafy et al., did not use ultrasound to directly guide extubation decisions, but just to assess tolerance during an SBT, and b) imprecision – as the 95% CI included a decision threshold of 0.75. We did not downgrade this finding due to risk of bias as only one of the three RCTs was considered at high risk of bias and this RCTs was statistically weighted very low in the analysis due to its small sample size.

**Table 2:** Summary of findings – diaphragmatic ultrasound guided extubation compared to standard criteria (without diaphragmatic ultrasound) for people who are on mechanical ventilation in intensive care units

Outcome No. of participants (studies)	Risk ratio (95% CI)	Anticipated absolute effects (95% CI)			Certainty
		Standard criteria	Diaphragmatic ultrasound	Difference	
Reintubation within 48 h No. of participants: 268 (3 RCTs)	**RR 0.62** (0.41 to 0.95)	31.6%	**19.6%** (12.9 to 30)	**12.0% fewer** (18.6 fewer to 1.6 fewer)	⨁⨁◯◯ Low^a,b^
Total duration of mechanical ventilation No. of participants: 268 (3 RCTs)	–	The mean total duration of mechanical ventilation was **110.7** h	–	MD **1.39 h lower** (17.5 lower to 14.71 higher)	⨁◯◯◯ Very low^a,c,d^
Reintubation after 48 h No. of participants: 240 (2 RCTs)	**RR 0.38** (0.11 to 1.29)	35.0%	**13.3%** (3.9 to 45.1)	**21.7% fewer** (31.1 fewer to 10.2 more)	⨁⨁⨁◯ Moderate^e^
Total ICU length of stay No. of participants: 130 (1 RCT)	–	The mean total ICU length of stay was **7.8** days	–	MD **1 days lower** (1.74 lower to 0.26 lower)	⨁⨁◯◯ Low^d,f^
Adverse event No. of participants: 0 (0 RCT)	Not estimable	0.0%	**0.0%** (0 to 0)	**0.0% fewer** (0 fewer to 0 fewer)	–

*The risk in the diaphragmatic ultrasound group (and its 95% confidence interval) is based on the assumed risk in the standard criteria group and the relative effect of the intervention (and its 95% CI) *CI* confidence interval; *MD* mean difference; *RR* risk ratio

a. Downgraded one level due to indirectness (the purpose of ultrasound use differed from that of the target context)

b. The 95% confidence interval of the risk ratio includes both the decision threshold of 0.75

c. Downgraded one level due to considerable heterogeneity (*I*^2^ = 76%)

d. Downgraded one level due to a smaller sample size than OIS

e. The 95% confidence interval of the risk ratio includes both the decision thresholds of 0.75 and 1.25

f. Downgraded one level due to crossing − 1 in the 95% confidence interval (the threshold for ICU length of stay was set at ± 1 day)

### Secondary outcomes

Three studies (268 participants) provided data on total duration of mechanical ventilation [24–26]. There was no difference in the total duration of mechanical ventilation between the diaphragmatic ultrasound and standard criteria groups (MD − 1.39 h; 95% CI − 17.5 to 14.71 h; very low certainty of evidence) (Fig. 3, Table 2). Two study (240 participants) provided data on the risk of reintubation after 48 h of extubation [22, 23]. These studies found that there was no difference in the risk of reintubation after 48 h between the diaphragmatic ultrasound and standard criteria groups (RR = 0.38; 95% CI 0.11 to 1.29; moderate certainty of evidence) (Fig. 3, Table 2). The other study (130 participants) provided data on length of ICU stay [25]. This study found that patients who received diaphragmatic ultrasound to guide liberation decisions spent one day less in ICU on average compared to people who received usual care (MD − 1.0 day; 95% CI − 1.74 days to − 0.26 days; low certainty of evidence) (Fig. 3, Table 2). No studies reported any adverse events.

## Discussion

This systematic review was conducted to compare reintubation rates after extubation for adults on mechanical ventilation where the decision to extubate was based on diaphragmatic ultrasound in addition to standard clinical criteria versus standard clinical criteria alone. As shown by the results of the meta-analysis, decision-making based on diaphragmatic ultrasound significantly reduced the risk of reintubation within 48 h of extubation. In addition, the use of diaphragmatic ultrasound to guide decisions around ventilator liberation was associated with reduced lengths of stay in ICU. However, no differences were found regarding total duration of mechanical ventilation or reintubation rates after 48 h of extubation.

Other systematic reviews conducted to date to evaluate the effectiveness of diaphragmatic ultrasound during mechanical ventilation liberation have focused on assessment of diagnostic accuracy [4, 8, 27]. These reports have demonstrated that measurements of DE and DTF have a high diagnostic accuracy for predicting successful liberation from mechanical ventilation. However, to the best of our knowledge, this is the first systematic review to focus on RCTs on the effectiveness of diaphragmatic ultrasound indices to improve success with the ventilator liberation process.

In this systematic review, using diaphragmatic ultrasound in addition to standard clinical criteria to guide decisions around ventilator use and liberation resulted in a reduced risk of reintubation within 48 h of extubation when compared to standard clinical criteria alone. This review analyzed three RCTs that used indicators such as DTF and DRSBI. Previous studies have shown that pre-extubation DTFs above 26–30% predict higher success rates with extubation [5, 28]. In this review, Toledo et al. and McCool et al. used DTF greater than 30% to guide decision-making, while Alansary et al. used DTF greater than 26% as the extubation indicator, employing criteria similar to those in prior studies. It has been reported that RSBI, a traditional predictive index of successful extubation, has decreased diagnostic accuracy due to involvement of the respiratory support muscles resulting from fatigue, potentially hiding diaphragmatic dysfunction [29], and that therefore diaphragmatic activity during extubation should be monitored instead. As a result, DRSBI has been proposed as an alternative for evaluating readiness for extubation, replacing RSBI’s tidal volume with diaphragmatic movement distance.

Previous studies have reported higher extubation success rates when the DRSBI is less than 1.38–1.77 breaths/minute/millimeter [7, 30]. Among the studies included in this review, Mowafy et al. used a DRSBI of less than 1.3 breaths/minute/millimeter as an extubation indicator, applying a similar criterion to previous studies. In predicting successful extubation, DTF has been shown to be more sensitive and specific than DE and is more accurate than DE or RSBI, while DRSBI is more diagnostically accurate than conventional RSBI [5, 27, 29, 30]. Therefore, the approaches to diaphragmatic ultrasound used in studies included in this systematic review were appropriate and appeared to contribute to a reduction in the risk of reintubation within 48 h of extubation.

This review has several limitations. First, because it was limited to RCTs, only five studies were included, with only three contributing data to the meta-analysis of the primary outcome. As a result, the findings of this review are based on analyses with low statistical power due to the small overall sample size. Second, statistical heterogeneity was observed in the data on mechanical ventilation duration. This may be due to differences in patient populations across the studies, with some involving medical conditions and others involving surgical conditions. In addition, the variations in diaphragmatic ultrasound indicators may have also contributed to heterogeneity. However, we were not able to identify enough studies to conduct subgroup analyses to test these or other possible assumptions about causes of this heterogeneity. Future studies may improve the precision of the estimates of effects sizes reported in this review or provide a better understanding of the causes of differences in extubation outcomes.

## Conclusions

Using diaphragmatic ultrasound in addition to standard clinical criteria to guide decisions around ventilator use and liberation resulted in a reduced risk of reintubation within 48 h of extubation when compared to standard clinical criteria alone. Further RCTs, considering different health conditions or severities of illness, are needed to improve our understanding of how best to use diaphragmatic ultrasound during ventilator liberation.

## Supplementary Information

Additional file 1.

Additional file 2.

## Data Availability

No datasets were generated or analyzed during the current study.

## Abbreviations

GRADE: Grading of Recommendations, Assessment, Development, and Evaluation
ICU: Intensive care unit
RR: Relative risk
CI: Confidence interval
RSBI: Rapid shallow breathing index
DTF: Diaphragm thickening fraction
DE: Diaphragmatic excursion
DRSBI: Diaphragmatic rapid shallow breathing index
RCT: Randomized controlled trial
SBT: Spontaneous breathing trial
ITT: Intention to treat
