# Supplementary material for: Clinical utility of diaphragmatic ultrasound for mechanical ventilator liberation in adults: a systematic review and meta-analysis
Source: J Intensive Care. 2025 Jul 24;13:40. doi: 10.1186/s40560-025-00811-0 (PMC12288223; doi:10.1186/s40560-025-00811-0)
Supplement: Supplementary file 1 — Additional file 1. [file 40560_2025_811_MOESM1_ESM.docx]

**Table S1. Literature search strategy for each database**

**PubMed search strategy (Search date; 1 April 2025)**

**CENTRAL search strategy (Search date; 1 April 2025)**

**EMBASE search strategy (Search date; 1 April 2025)**
