# Supplementary material for: Clinical utility of diaphragmatic ultrasound for mechanical ventilator liberation in adults: a systematic review and meta-analysis
Source: J Intensive Care. 2025 Jul 24;13:40. doi: 10.1186/s40560-025-00811-0 (PMC12288223; doi:10.1186/s40560-025-00811-0)
Supplement: Supplementary file 2 — Additional file 2. [file 40560_2025_811_MOESM2_ESM.docx]

Total duration of mechanical ventilation

Reintubation after 48 h

Total ICU length of stay

Additional File 2: Figure S1. Summary of risk of bias for secondary outcomes.
